# Screening for familial hypercholesterolaemia in childhood: Avon Longitudinal Study of Parents and Children (ALSPAC)

**DOI:** 10.1016/j.atherosclerosis.2017.03.007

**Published:** 2017-05

**Authors:** Marta Futema, Jackie A. Cooper, Marietta Charakida, Christopher Boustred, Naveed Sattar, John Deanfield, Debbie A. Lawlor, Nicholas J. Timpson, UK10K Consortium, Steve E. Humphries, Aroon D. Hingorani

**Affiliations:** a Centre for Cardiovascular Genetics, Institute of Cardiovascular Science, University College London, London, UK; b National Centre for Cardiovascular Prevention and Outcomes, Institute of Cardiovascular Science, University College London (UCL), London, UK; c North East Thames Regional Genetics Service, Great Ormond Street Hospital for Children, London, UK; d Institute of Cardiovascular and Medical Sciences, British Heart Foundation Glasgow, Cardiovascular Research Centre, University of Glasgow, Glasgow, UK; e MRC Integrative Epidemiology Unit at the University of Bristol, Bristol, UK; f School of Social and Community Medicine, University of Bristol, Bristol, UK; g UCL Genetics Institute, Department of Genetics, Environment and Evolution, University College London, London, UK; h Genetic Epidemiology Group, Institute of Cardiovascular Science, Farr Institute for Health Informatics, University College London, London, UK

**Keywords:** ALSPAC, Familial hypercholesterolaemia, LDL-cholesterol, Next generation sequencing, Familial hypercholesterolaemia screening, Total cholesterol

## Abstract

**Background and aims:**

Familial hypercholesterolaemia (FH) is an autosomal-dominant disease with frequency of 1/500 to 1/250 that leads to premature coronary heart disease. New approaches to identify FH mutation-carriers early are needed to prevent premature cardiac deaths. In a cross-sectional study of the Avon Longitudinal Study of Parents and Children (ALSPAC), we evaluated the biochemical thresholds for FH screening in childhood, and modelled a two-stage biochemical and sequencing screening strategy for FH detection.

**Methods:**

From 5083 ALSPAC children with cholesterol measurement at age nine years, FH genetic diagnosis was performed in 1512 individuals, using whole-genome or targeted sequencing of known FH-causing genes. Detection rate (DR) and false-positive rate (FPR) for proposed screening thresholds (total-cholesterol > 1.53, or LDL-C > 1.84 multiples of the median (MoM)) were assessed.

**Results:**

Six of 1512 sequenced individuals had an FH-causing mutation of whom five had LDL-C > 1.84 MoM, giving a verification-bias corrected DR of 62.5% (95% CI: 25–92), with a FPR of 0.2% (95% CI: 0.1–0.4). The DR for the TC cut-point of 1.53 MoM was 25% (95% CI: 3.2–65.1) with a FPR of 0.4% (95% CI: 0.2–0.6). We estimated 13 of an expected 20 FH mutation carriers (and 13 of the 20 parental carriers) could be detected for every 10,000 children screened, with false-positives reliably excluded by addition of a next generation sequencing step in biochemical screen-positive samples.

**Conclusions:**

Proposed cholesterol thresholds for childhood FH screening were less accurate than previously estimated. A sequential strategy of biochemical screening followed by targeted sequencing of FH genes in screen-positive children may help mitigate the higher than previously estimated FPR and reduce wasted screening of unaffected parents.

## Highlights

• 6 FH mutations were found in ALSPAC children cohort by next generation sequencing.
• Proposed children LDL cut-offs resulted in higher than expected false positive rate.
• Sequencing step could improve the efficiency of parental FH screening.

## 1 Introduction

Familial hypercholesterolaemia (FH) is an inherited disorder with prevalence estimated from recent epidemiological[1] and sequencing studies[2], [3] of 1 in 250, higher than historical estimates of 1 in 500. Autosomal dominant FH is caused by mutations in genes encoding the low-density lipoprotein receptor (*LDLR*) [4], apolipoprotein B (*APOB*) [5], and proprotein convertase subtilisin/kexin type 9 (*PCSK9*) [6]. Autosomal recessive FH is caused by mutations in the *LDLRAP1* gene[7]. Characteristics include elevated total cholesterol (TC) and LDL-C, cutaneous lipid deposition, and a high risk of premature coronary heart disease (CHD). Untreated FH patients exhibit a 13-fold excess risk of CHD compared to the general population[1], with FH men typically developing CHD in their 50s, and women in their 60s[8]. European guidelines recommend early high potency statin treatment to achieve maximal LDL-C reduction[9].

Pre-clinical screening of first-degree relatives of a patient (cascade testing) has been carried out effectively in Europe[10], [11]. However, index cases are usually ascertained opportunistically (at clinical presentation) rather than systematically. Worldwide, fewer than 5% of FH individuals have a diagnosis, with only 12% known and treated in the UK [9].

New approaches are needed to identify affected individuals before they develop CHD. In 2007, Wald et al. proposed biochemical FH screening by measurement of TC and LDL-C in childhood (one to nine years of age), when the separation of the lipid distribution in affected and unaffected is greater than later in life[12]. The detection rate (DR), estimated for an LDL-C cut-point of 1.84 multiples of the median (MoM), or a TC of 1.53 MoM was 85% or 88% respectively, for a false positive rate (FPR) of 0.1%. It was estimated the affected parent could then be detected as the one with the higher cholesterol, with an FPR of 4%. A preliminary report based on ∼200 children screened at the age of 15 months was published in 2011 [13], and the outcome of a 10,000 child screening study was reported in 2016[14].

However, in the primary report[12], the proposed biochemical thresholds were based on historic case-control data: lipid values in FH cases were ascertained from hospital clinic records, while those in controls were from unaffected siblings of patients, who may have been from a different age stratum to the cases, or from values in healthy population surveys from the same geographical region, conducted within 5 years of the case ascertainment. In addition, FH was confirmed either by clinical criteria or by mutation-detction methods that predated the more sensitive next generation DNA sequencing (NGS).

In the outcome study[14], a more relaxed case definition was adopted than in the prior meta-analysis of case-control data. A case was defined as *either* carriage of an FH mutation *or* a persistently high cholesterol, which risks mixing polygenic hypercholesterolaemia with monogenic FH. Using cholesterol both in the test and case definition also complicates assessment of screening performance. Finally, a 48 variant mutation detection panel was used rather than a sequencing approach, which runs the risk of missing cases with FH mutations not represented in the panel.

We, therefore, evaluated the performance of previously proposed LDL-C and TC thresholds for the detection of FH in a general population sample of children from the Avon Longitudinal Study of Parents and Children (ALSPAC) [15], using NGS of FH genes as the diagnostic standard.

## 2 Materials and methods

ALSPAC study description and the ethical approval are presented in the Supplementary Material.

### 2.1 Cholesterol measurement

At a mean (standard deviation, SD) age of 9.9 years (4 months), TC, LDL-C (calculated using the Friedewald equation) and other lipids and apolipoproteins were measured in non-fasting blood samples from 5083 ALSPAC children, using methods described previously[16]. Non-high-density-lipoprotein cholesterol (non-HDL-C) was calculated by subtracting HDL-C from TC.

### 2.2 DNA sequencing for FH-causing mutations

A 30% random sample of ALSPAC participants (N = 1503) were previously selected and 1497 (29.5%) successfully completed low-read depth whole genome sequencing (WGS, see Supplementary Material), as part of the UK10K project[3].

**Fig. 1:**
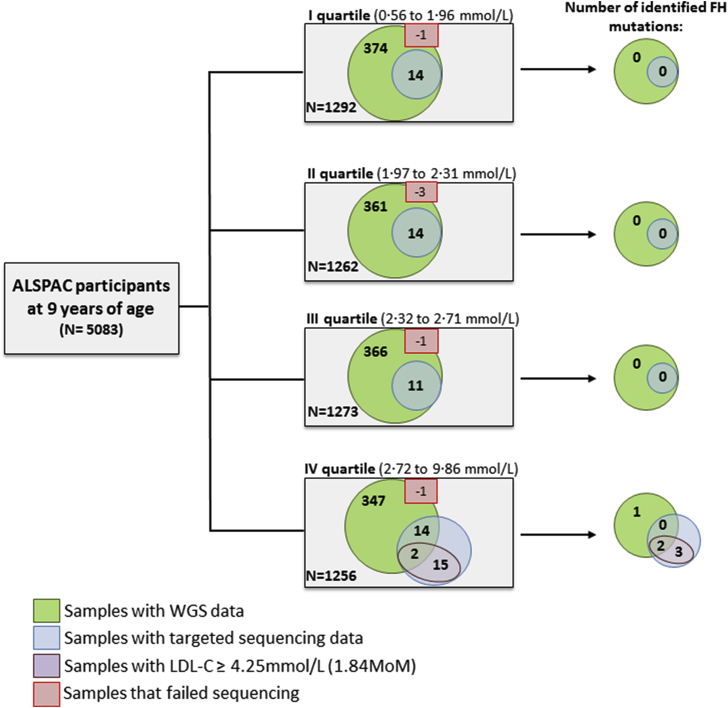
Study design. Of 5083 ALSPAC samples with lipids measured at the age of nine years, 1497 were successfully sequenced as part of the UK10K project. 55 of these, selected by stratified random sampling from each LDL-C quartile, underwent targeted sequencing (*LDLR*, *APOB*, *PCSK9, LDLRAP1*) together with 15 samples from children with LDL-C>1.84 MoM or TC > 1.53 MoM. Six FH-causing mutations were identified. Six samples failed NGS sequencing.

In addition, we conducted targeted high-read depth sequencing (see Supplementary Material) of the known FH genes (*LDLR*, *APOB*, *PCSK9,* and *LDLRAP1*) in 55 samples selected by stratified random sampling from each quartile of the LDL-C distribution, restricting to those that were also included in the UK10K project. A further 15 samples with an LDL-C>1.84 MoM, who also had a TC > 1.53 MoM, were selected for targeted NGS, giving a total of 70 with targeted sequencing data (Fig. 1), and 1512 samples with any sequencing data. No other clinical characteristics were included in the selection of these individuals. The variant interpretation methods are shown in the Supplementary Material.

### 2.3 Genotyping of common LDL-C-raising alleles

Since carriage of a high burden of common LDL-C-raising alleles can mimic the biochemical features of monogenic FH[17], [18], and might contribute to a false positive FH diagnosis, we also obtained genotypes for six LDL-C-associated SNPs: rs629301 (*CELSR2*), rs1367117 (*APOB*), rs6544713 (*ABCG8*), rs6511720 (*LDLR*), rs429358 (*APOE*), rs7412 (*APOE*), in the whole cohort. The methods used for genotyping, generating and validating a weighted LDL-C genetic risk score were described previously[17], [18].

### 2.4 Statistical analysis

Data were analysed using R (http://www.r-project.org) and WINPEPI[19]. We compared categorical and continuous variables using χ^2^ and two sample t-tests respectively, generating *p*-values on the basis of the null hypothesis of no difference between the groups. To evaluate the performance of previously proposed LDL-C (1.84 MoM, 1.66 MoM and 1.58 MoM) and TC cut-offs (1.53 MoM, 1.42 MoM and 1.37 MoM) [12], we obtained estimates of DR, FPR, predictive value of a positive test (PPV), predictive value of a negative test (NPV), and odds of being affected given a positive test[20]. Since all samples with LDL-C >1.84 MoM or TC > 1.53 MoM underwent targeted sequencing but only a proportion of samples with LDL-C or TC below these values was sequenced, the study design was subject to verification bias. Information on the number of participants with FH mutations in the sampled group with LDL-C/TC below the pre-specified cut-points was therefore used to estimate the prevalence of FH mutations in the whole cohort by scaling. 95% confidence intervals were based on binomial probabilities.

The performance of the child-parent screening approach was evaluated with and without adjustment for verification bias and compared with previous estimates[12], [14]. We also estimated the effect of misclassification of case status arising from imperfect accuracy of NGS screening performance, based on a 90% rather than 100% sensitivity of NGS, following the method of Greenland et al.[21]. The FPR of NGS was assumed to be equal to 0%, because all samples with mutations identified by NGS undergo confirmation by Sanger sequencing.

## 3 Results

Mean (SD) and median (interquartile range) values of LDL-C, TC, and a range of other variables were similar between the 1512 children who successfully underwent whole genome, targeted sequencing, or both, and the 3571 who did not (Supplementary Table 1).

All regions of the known FH genes were sequenced at an average of 7 × read depth in the UK10K WGS sub-sample[3] and, with the exception of exon 2 in *APOB*, at a minimum of 15 × read depth in the targeted sequencing sub-sample (Supplementary Fig. 1). The overall coverage of *LDLRAP1* was insufficient (Supplementary Fig. 1) therefore analysis of variants in this gene, associated with the rare recessive FH, was excluded. An FH-causing mutation was identified in six individuals (Table 1). Four of the identified mutations located in *LDLR* had previously been reported as being FH-causing[22], [23], and were predicted to be ‘damaging’ using *in silico* programmes. Two children carried the most common FH mutation, p(Arg3527Gln), located in *APOB*. Compared to the children who underwent sequencing but in whom no FH mutation was identified (n = 1506), FH mutation carriers had a higher LDL-C (mean (SD): 4.72 (1.35) mmol/L *vs.* 2.34 (3.14) mmol/L, *p* = 0.02), TC (6.34 (1.32) mmol/L *vs.* 4.27 (0.72) mmol/L, *p* = 0.03), and apolipoprotein B (104.3 (30) mg/dL *vs*. 58.5 [13]mg/dL, *p* = 0.03) (*p* = 0.02) (Supplementary Table 3).

**Fig. 2:**
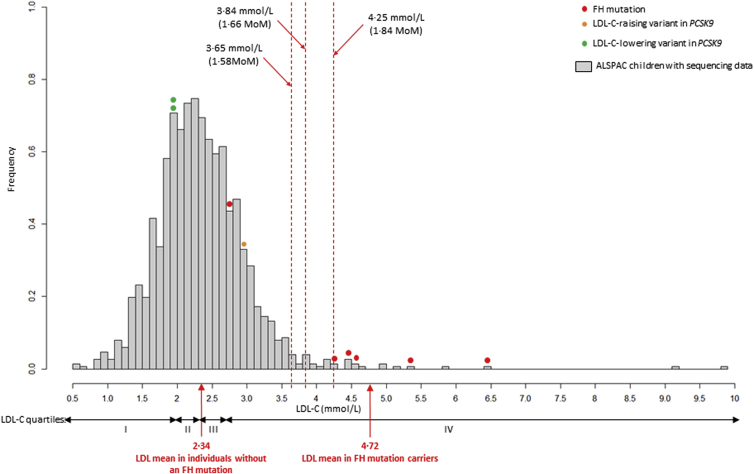
LDL-C distribution at 9 years of age in sequenced children. Red dashed lines indicate FH diagnostic LDL-C cut-points (1.84 MoM, 1.66 MoM, and 1.58 MoM). Five of six mutation carriers (marked red) had LDL-C above the cut-points. Two children carried a loss-of-function variant (p.Arg46Leu) in the *PCSK9* gene, known to lower LDL-C (marked green) [6], [26]. One child was found to have a rare *PCSK9* variant (p.His553Arg) associated with increased LDL-C (marked orange) [27]. (For interpretation of the references to colour in this figure legend, the reader is referred to the web version of this article.)

Median LDL-C in the whole sample was 2.3 mmol/L, yielding FH diagnostic cut-points of 4.25 mmol/L (1.84 MoM for an estimated FPR of 0.1%), 3.84 mmol/L (1.66 MoM for an estimated FPR 0.5%), and 3.65 mmol/L (1.58 MoM for an estimated FPR 1%) (Fig. 2). Median TC was 4.23 mmol/L yielding TC cut-points of 6.47 mmol/L (1.53 MoM; for an estimated FPR of 0.1%), 6.01 mmol/L (1.42 MoM; estimated FPR 0.5%), and 5.80 mmol/L (1.37 MoM; estimated FPR 1%).

For all three LDL-C cut-points, based on the sequenced sample alone, the observed DR was 83% (95% CI: 36–100), lower than the estimated DRs of 85%, 93%, and 96%, respectively, from historic data[12]. The corresponding FPRs were also higher at 0.8% (95% CI: 0.4–1.4), 1.53% (95% CI:1.08–2.44), and 2.3% (95% CI: 1.6–3.2), respectively (Table 2A; Supplementary Table 4A). FH screening using TC was less accurate than LDL-C, shown in Table 2B, and Supplementary Table 4B.

The study design introduced the potential for verification bias because we exhaustively sequenced individuals with LDL-C>1.84 MoM, but only a stratified random sample of individuals with LDL-C below this threshold. Among the 1495 sequenced participants with LDL-C<1.84 MoM, we identified one with an FH mutation. We therefore re-estimated the childhood LDL-C screening performance on the assumption that among all 5066 individuals with LDL-C<1.84 MoM either there would be no further FH mutations or, under the more conservative assumption, that there would be a total of 5066/1495 (∼3) individuals with a mutation (see Supplementary Table 6 for calculations). The corresponding estimates of the screening performance adjusted for verification bias are shown in Table 2 column III. The DR for an LDL-C of 1.84 MoM, on the assumption of no further false negatives was 83% (95% CI:35.9–99.6), with an FPR improvement to 0.2% (95% CI: 0.1–0.4). However, after adjustment for verification bias, the DR fell to 62.5% (95%CI: 24.5–91.5) with an FPR of 0.2% (95% CI: 0.1–0.4). Further scenarios were modelled where the sequence DR for FH-causing mutations was either 100% or 90%, the latter being an extremely conservative lower boundary based on the performance of different NGS methods[3], [24], [25]. The effect of imperfect accuracy of NGS on screening performance is shown in Table 2, column IV.

The TC threshold of 1.53 MoM had a lower DR of 33% (95%CI: 4.3–77.7), falling to 25% (95% CI: 3.2–65.1), after adjustment for verification bias. DR improved with lower cut-points at the expense of a higher FPR (Supplementary Table 4B). Detection rate of FH mutation carriers using non-HDL-C cut-offs was very similar to the DR produced by TC cut-offs, with slightly lower FPR (Supplementary Table 3C).

The mean (SD) predicted LDL-C weighted gene score based on six SNPs was 0.62 (0.23) mmol/L (Supplementary Fig. 2), which did not differ from the previously studied Whitehall II sample (mean 0.63 (0.22) mmol/L), where a 1 SD increase in the score was associated with a 0.33 (SE = 0.02) mmol/L increment in LDL-C(17). Individuals with LDL-C>1.84 MoM but with no FH mutation had a higher predicted LDL-C gene score (0.72(0.17) mmol/L) than those with LDL-C<1.84 MoM (0.63 (0.23) mmol/L, *p* = 0.09). The mean score in those who were found to be FH mutation positive was lower than in those with an LDL-C>1.84 MoM but with no mutation identified (0.59 (0.21) mmol/L *vs.* 0.72 (0.17) mmol/L, *p* = 0.23, consistent with previous findings(17, 18)). All but one (91.7%) of those with LDL-C>1.84 MoM and no FH mutation detected had predicted LDL-C weighted gene score above the bottom quartile, of whom 42% had score in the top quartile, which suggests polygenic hypercholesterolaemia as an alternative diagnosis in these individuals.

**Fig. 3:**
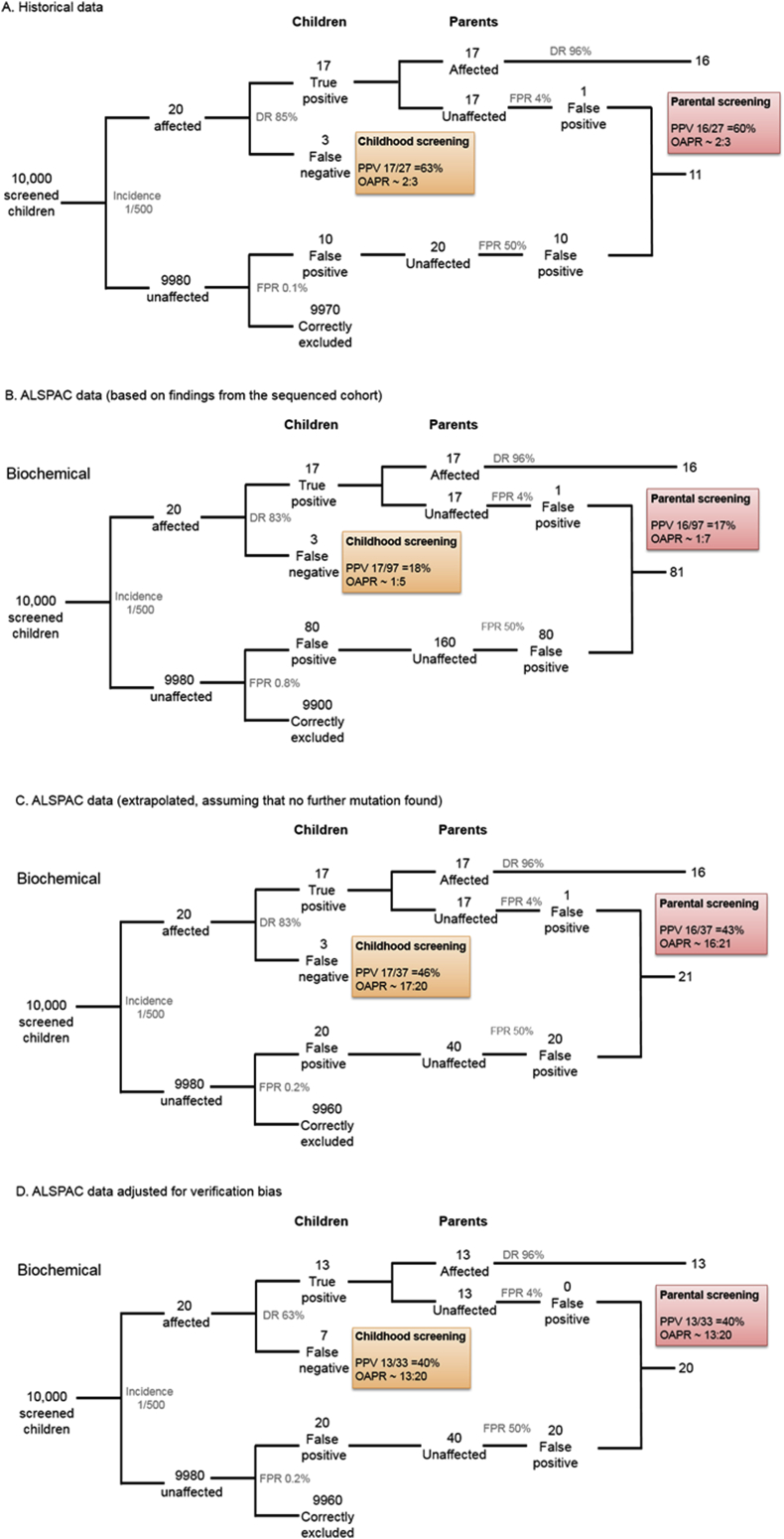

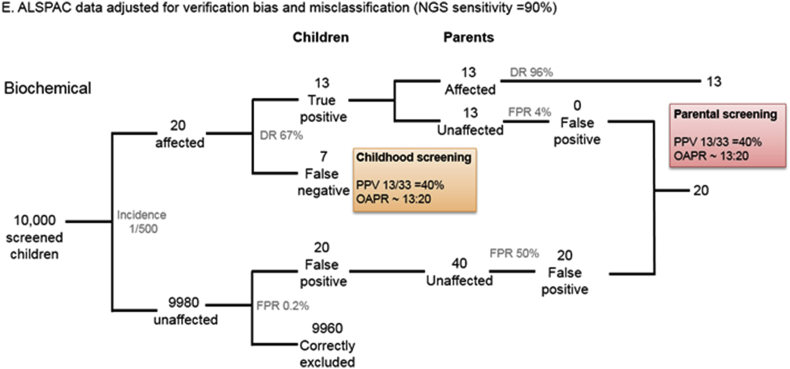
Estimated performance of child-parent FH screening. Estimated performance of child-parent FH screening in a hypothetical sample of 10,000 children and their parents, assuming a prevalence of FH 1 in 500, based on: (A) the observed performance of LDL-C (1.84 MoM) compared with the historical data [12]; (B) DR and FPR in the sequenced sample; (C) extrapolation to the whole ALSPAC, assuming that no further mutations would be found; (D) extrapolation accounting for verification bias; (E) extrapolation corrected for verification bias and NGS sensitivity of 90%. (DR = detection rate, FPR = false positive rate, PPV = positive predictive value, OAPR = odds of being affected given a positive result).

Information on FH mutations and LDL-C in the parents of the children studied was unavailable. However, we estimated the likely accuracy of the child-parent screening based on the observed performance of 1.84MoM LDL-C cut-point in ALSPAC and compared it with the original report by Wald and colleagues. As shown in Fig. 3A, **i**n a hypothetical sample of 10,000 children, in which the prevalence of FH is 1 in 500, and using the Wald et al. estimates for the DR and the FPR for biochemical screening of 85% and 0.1%, respectively, we would expect to find 17 true positives (TP) and 10 false positives (FP), giving an estimated PPV of 63%. Using the assumption of Wald et al. that the affected parent is the one with the higher LDL-C, which was estimated to give a parental FPR of 4%, the PPV for parental screening would be 60% (Fig. 3A). However, if the DR was actually 83% and the FPR was 0.8%, as seen in the ALSPAC sequenced subsample alone, FPs would greatly outnumber TPs (80 FP children to 17 TPs) with a consequent fall in PPV (Fig. 3B). On the other hand, if the assumption holds that there are no additional FH-mutation carriers among those with LDL-C<1.84MoM, the DR would be preserved but the FPR would fall to 0.2% (Fig. 3C). If however, the true screening performance of an LDL-C cut-point of 1.84MoM is closer to that estimated after adjustment for verification bias, i.e. a DR of 62.5% with a FPR of 0.2%, we estimated the PPV of the 1.84MoM LDL-C threshold would fall to 40% in both children and parents (Fig. 3D). Misclassification of case status due to NGS having a 90% rather 100% DR for FH mutation had little additional consequence (Fig. 3E).

Under all scenarios, the addition of an NGS step for samples from all children who screen positive on the basis of an LDL-C, would eliminate the FPs, if the DR and FPR of NGS are close to 100% and 0%, respectively, and reduce the rate of misclassification among the parents, because parents of children with false-positive biochemical screening would be eliminated from subsequent evaluation. Table 3 summarises the number of FH children and affected parents identified for every 10,000 children screened, under the range of scenarios discussed, with and without the interpolation of an NGS step. An illustration of the screening process is shown in Supplementary Fig. 3.

## 4 Discussion

We evaluated the performance of a previously proposed biochemical screening strategy for FH [12] in a large general population sample of 5083 nine-year old UK children, using comprehensive NGS methods for diagnosis of FH. Based on a sequenced subsample of 1512 children, an LDL-C threshold of 1.84 MoM (4.25 mmol/L) led to a DR of 83%, close to that previously estimated, but at a higher FPR (0.8%; 95% CI: 0.4–1.4%) than the pre-specified 0.1% [12].

We re-estimated the screening performance of the proposed LDL-C threshold after extrapolation to the whole cohort for two extreme scenarios. In the first, the assumption was made that no further individuals with FH mutations would be found among those with LDL-C<1.84 MoM. In the other, it was assumed that a total of three FH mutation carriers (rather than one) would be expected among those with an LDL-C<1.84 MoM, thereby accounting for verification bias. The first assumption yielded a DR for childhood screening of 83% with an FPR of 0.2%, much closer to that estimated by Wald and colleagues. The second led to a much lower DR (62.5%) with a similar FPR (0.2%) to the historical data. Thus, depending on the assumptions, the accuracy of childhood screening for FH estimated from historic case-control data could underestimate the FPR or overestimate the DR, both of which would affect the efficiency of parental screening.

We next modelled the effect of including an NGS step in all screen positive samples, followed by the exclusion of parents of children where sequencing did not confirm a mutation (false-positives). Because of the very high DR and low FPR of NGS this would have the benefit of effectively eliminating parents of false-positive children from the screening programme. The performance of such a programme would then depend on the number of affected individuals ascertained which, in turn, depends on the DR of biochemical screening.

In the best case scenario, with a DR of around 85%, followed by NGS to eliminate false-positive samples, we estimated that 16 or 17 children and a similar number of parents (32–34 in total) would be detected from the 40 parents and children expected to be affected for every 10,000 children and 20,000 parents screened, assuming a prevalence of 1 in 500. If the DR from biochemical screening is closer to 63%, then 13 children and 13 parents (a total of 26 out of 40 affected individuals) would be ascertained. Estimates based on the TC were markedly lower than for LDL-C.

There are several possible reasons for the higher biochemical FPR in ALSPAC than that previously estimated[12]. Estimates of the accuracy of biochemical screening of FH were previously derived from studies in which children were 1–9 years of age. We measured LDL-C at an average age of 9.9 years. The overlap of LDL-C between affected and unaffected at this age may be higher than at a younger age, leading to a higher FPR. The higher FPR in ALSPAC may also simply reflect the play of chance, because of the small absolute number of FH cases observed in a general population sample of this size (six FH cases in a subsample of 1512 children, compared to 253 FH cases in the pooled analysis reported previously[12]). Changes in diet and lifestyle may also have led to LDL-C in children being different than in the period over which prior case-control studies were reported. Controls in the historical data had higher median LDL-C than the ALSPAC cohort (*p* < 1 × 10^−4^). This environmental influence could affect the screening performance if it differentially influenced LDL-C in children who carry a high burden of common, small-effect LDL-C-raising alleles. We noted in the ALSPAC sample (n = 5083) that the average number of common LDL-C-raising alleles increased by the LDL-C quartile, but we did not have the power in our sample to investigate a gene-environment interaction. Alternatively, it may be that the published FPR estimate is lower than could be achieved by a childhood screening programme. In that analysis, cases were identified from lipid clinics, while controls were unaffected siblings, or healthy individuals from general population surveys, and this might have accentuated differences in LDL-C between cases and controls. Siblings of FH children might have been exposed to healthier diets and lifestyles as a response to having an FH diagnosis in the same family, while participants in population surveys may have been healthier on average than those who decline participation. The lower DR and higher FPR observed in ALSPAC than when compared to the original report is also consistent with the findings from the recent 10,000 child screening project[14].

If FPR estimates are higher than previously estimated, this would adversely affect the accuracy of biochemical screening of children and their parents, as illustrated in Fig. 3. However, the higher than estimated FPR of biochemical screening in childhood could be restored by NGS analysis of all biochemical screen positive samples (Table 3). The accuracy, cost-effectiveness and acceptability of such an approach, as well as the performance of alternative sequencing approaches (whole genome, whole exome or targeted sequencing), different sequencing technologies and bioinfomatic pipelines for sequence interpretation, would require separate study. Moreover, some individuals with an FH-causing mutation may not present with LDL-C levels over the specified cut-off because they have also inherited one or more common LDL-C-lowering variants, for example the p. Arg46Leu loss-of-function variant in *PCSK9*, thus inclusion of such known variants in the sequencing step should be considered.

To fully assess the performance of child-parent screening, samples from all mothers and fathers would have been available but, unfortunately, this was not the case. We would also like to have been able to sequence all samples from the ALSPAC study but this was precluded by the currently high cost of sequencing. We exploited the availability of information from low-read depth sequencing from 1503 samples included in the UK10K project, but were only able to conduct targeted NGS in a stratified random subsample drawn from all four quartiles of the LDL-C distribution, boosted by sequencing all samples from children with an LDL-C>1.84MoM. This design introduced the potential for verification bias, which we accounted for as described above. Some further technical limitations to our study are discussed in the Supplementary Material section.

In conclusion, an LDL-C cut-point of 1.84MoM for the biochemical screening in childhood could be inaccurate unless performed soon either because of underestimation of the FPR or overestimation of the DR. The performance of biochemical screening for FH in childhood might be improved by assessment at an age younger than 9 years, and/or by incorporating a sequential NGS step to exclude false positives, and eliminate wasted screening in the parental generation.

## Conflict of interest

NS has relationships with AMGEN, Sanofi and Merc, MF has received a honorarium from Sanofi for a talk not related to the article, and AH has non-financial relationships with Illumina that might have an interest in the submitted work in the previous 3 years,MF, JAC, MC, CB, DL, JD, SHE, NJT, AH have no non-financial interests that may be relevant to the submitted work.

## Financial support

The funding bodies of the study had no role in study design, data collection, data analysis, data interpretation, or writing of the report. The corresponding author had full access to all the data in the study and had final responsibility for the decision to submit for publication.

## Author contributions

Authors MF, NS, JD, DAL, NJT, SHE and ADH contributed to the conception and design of the work. Authors MF, JAC, MC, CB acquired the data and performed the analysis. All authors were involved in the interpretation of data. MF, SHE and ADH drafted the work, which was critically revised by all other authors. All authors approved the final version of the manuscript.

## Figures and Tables

**Table 1:** FH mutations identified in the study.

Gene	DNA:Protein change	LDL-C of the carrier (mmol/L) (percentile)	TC of the carrier (mmol/L) (percentile)	Mutation Predictions		
				PolyPhen-2	SIFT	Mutation Taster
**FH mutations**						
*LDLR*	c.680_681delAC:p.(Asp227Glyfs*12)	6.48(99th)	7.83(99th)	*Frame shift mutation*		
	c.722T > C:p.(Phe241Ser)	4.47(99th)	5.63(97th)	PD	NT	DC
	c.940G > A:p.(Gly314Arg)	2.79(80th)	4.61(72nd)	*Likely to affect splicing*		
	c.1897C > T:p.(Arg633Cys)	5.36(99th)	7.46(99th)	PD	NT	DC
*APOB*	c.10580G > A:p.(Arg3527Gln)	4.27(99th)	6.38(99th)	PD	NT	DC
		4.52(99th)	6.17(99th)	PD	NT	DC

Full characteristics of the variant carriers are shown in Supplementary Table 2.

PD = probably damaging; PsD = possibly damaging; NT = not tolerated; T = tolerated; DC = disease causing; P = polymorphism.

**Table 2:** Assessment of the biochemical screening for FH based on: (A) LDL-C, (B) TC, at nine years of age.

A.					
LDL-C	I	II	III	IV	
**1·84 MoM (4.25 mmol/L)**					Wald et al. 2007
DR (95%CI)	83% (35.9 to 99.6)	83% (35.9 to 99.6)	62.5% (24.5 to 91.5)	66.7% (29.9 to 92.5)	85% (79 to 89)
FPR (95%CI)	0.8% (0.4 to 1.4)	0.2% (0.1 to 0.4)	0.2% (0.1 to 0.4)	0.2% (0.1 to 0.4)	0.1%
PPV (95%CI)	29.4% (10.3 to 56.0)	29.4% (10.3 to 56.0)	29.4% (10.3 to 56.0)	35.3% (14.2 to 61.7)	NA
NPV (95%CI)	99.9% (99.6 to 100)	99.9% (99.9 to 100)	99.9% (99.6 to 99.9)	99.9% (99.8 to 100)	
OAPR (95%CI)	5:12 (0.15 to 0.72)	5:12 (0.15 to 0.72)	5:12 (0.15 to 0.72)	6:11 (0.23 to 0.83)	
B.					
TC	I	II	III	IV	
**1·53 MoM (6.47 mmol/L)**					Wald et al. 2007
DR (95%CI)	33% (4.3 to 77.7)	33% (4.3 to 77.7)	25% (3.2 to 65.1)	22.2% (2.8 to 60.0)	88% (84 to 92)
FPR (95%CI)	0.9% (0.5 to 1.6)	0.3% (0.15 to 0.5)	0.4% (0.2 to 0.6)	0.4% (0.2 to 0.6)	0.1%
PPV (95%CI)	12.5% (1.6 to 38.3)	12.5% (1.6 to 38.3)	9.1% (1.1 to 29.2)	9.1% (1.1 to 29.2)	NA
NPV (95%CI)	99.7% (99.3 to 99.9)	99.9% (99.8 to 100)	99.9% (99.7 to 100)	99.9% (99.7 to 99.9)	
OAPR (95%CI)	2:14 (0.02 to 0.43)	2:14 (0.02 to 0.43)	2:20 (0.01 to 0.32)	2:20 (0.01 to 0.32)	

The data analysis were based on: I, sequenced participants only; II, extrapolation to the whole cohort (n = 5083), assuming that there were no further FH mutations present in the not-sequenced participants; III, correction for verification bias; IV, correction for verification bias and misclassification based on a reduced sensitivity of NGS (90%). All values are shown in Supplementary Table 5 and Supplementary Table 6.

MoM = multiple of the median. DR = detection rate. CI = confidence intervals. FPR = false positive rate. PPV = positive predictive value. NPV = negative predictive value. OAPR = odds of being affected given a positive test. NA = not available.

**Table 3:** Comparison of the number of correctly identified and misclassified FH individuals in a hypothetical sample of 10,000 children, between the historical data(13) and the current study.

	Predicted cases based on the frequency of 1 in 500	Historical data	ALSPAC data			
			Sequenced data only	Extrapolated (if no further mutations found)	Extrapolated (adjusted for verification bias)	Extrapolated (adjusted for verification bias and NGS misclassification)
		DR = 85% FPR = 0.1%	DR = 83% FPR = 0.8%	DR = 83% FPR = 0.2%	DR = 63% FPR = 0.2%	DR = 66.7% FPR = 0.2%
		ID: M/C	ID: M/C	ID: M/C	ID: M/C	ID: M/C
Children (N = 10,000)	20	17: 10	17: 80	17: 20	13: 20	13: 20
Parents (N = 20,000)	20	16: 11	16: 81	16: 21	13: 20	13: 20
*If initial biochemical screening was followed by NGS*						
Children (N = 10,000)	20	17: 0	17: 0	17: 0	13: 0	13: 0
Parents (N = 20,000)	20	17: 0	17: 0	17: 0	13: 0	13: 0

Under all scenarios, the interpolation of a NGS for samples from all children who screen positive on the basis of an LDL-C above the diagnostic threshold, would reduce the misclassification rate to 0%.

DR = detection rate; FPR = false positive rate; NGS = next generation sequencing; ID: M/C = ratio of identified to misclassified.

## Appendix A Supplementary data

Athero FH in ALSPAC Supplementary data Athero FH in ALSPAC Supplementary data
