# Supplementary material for: Screening for familial hypercholesterolaemia in childhood: Avon Longitudinal Study of Parents and Children (ALSPAC)
Source: Atherosclerosis. 2017 May;260:47–55. doi: 10.1016/j.atherosclerosis.2017.03.007 (PMC5414415; doi:10.1016/j.atherosclerosis.2017.03.007)
Supplement: Athero FH in ALSPAC Supplementary data [file mmc1.docx]

**SUPPLEMENTARY MATERIAL**

**Screening for familial hypercholesterolaemia in childhood: Avon Longitudinal Study of Parents and Children (ALSPAC).**

Marta Futema, research associate^1^, Jackie A Cooper, research associate^1^, Marietta Charakida, clinician^2^, Christopher Boustred, bioinformatician^3^, Naveed Sattar, professor^4^, John Deanfield, professor and director^2^, Debbie A Lawlor, professor^5,6^, Nicholas J Timpson, reader^5,6^, UK10K Consortium^7^, Steve E Humphries, professor and director^1, 8*^, Prof Aroon D Hingorani, professor and director^9*^

**SUPPLEMENTARY METHODS**

**Study participants and cholesterol measurement methods are shown in the Appendix.**

ALSPAC recruited 14,541 women resident in Avon in the South West of England during pregnancy with expected dates of delivery of 1st April 1991 to 31st December 1992, with their partners and, subsequently their 13,988 children, who were live births and remained alive at 1 year of age([1](#_ENREF_1)). The fully searchable study data dictionary is found at http://www.bris.ac.uk/alspac/researchers/data-access/data-dictionary/. Approval for the study was obtained from the ALSPAC ethics and law committee, and written informed consent was obtained from the child’s main caregiver with assent to participate in each part of the study from the child.

**Whole Genome Sequencing (WGS)**

Low read-depth (average 6.7×) WGS data were obtained from the UK10K project (<http://www.uk10k.org/>). The sequencing protocol and the data production were previously described([2](#_ENREF_2)). The quality of the UK10K WGS data was previously analysed and was shown to be highly concordant with whole exome deep sequencing([3](#_ENREF_3)).

**Targeted Sequencing**

Primers to amplify coding regions (±25 base pairs (bp)) of the three autosomal dominant FH genes (*LDLR*, *APOB*, *PCSK9*), and the autosomal recessive FH gene (*LDLRAP1*), and six LDL-C-associated SNPs for targeted sequencing were designed using the Illumina DesignStudio. The length of amplicons was set to 250bp. The library preparation was performed using the TruSeq Custom Amplicon (TSCA) v1.5 kit (Illumina, San Diego, CA) and sequencing (in both directions) was done using the Illumina MiSeq platform.

Our criteria for standard variant calling pipeline were: coverage ≥30×, minimum of five reads for an altered allele, Phred quality ≥20, and a strand bias filter. To ensure that variants were not missed a sensitive pipeline was used (coverage ≥15×, minimum of two reads for an altered allele, Phred quality ≥ zero, no strand bias filter).

Copy number variants (CNVs) were called using the ExomeDepth package([4](#_ENREF_4)). All variants identified by the targeted sequencing were confirmed by Sanger sequencing.

Seven out of the 70 samples sequenced by the custom targeted sequencing method failed the sequencing process (due to insufficient DNA quality), however the majority of them (n=6) were successfully sequenced by WGS.

**Rare variant interpretation**

Common variants (frequency >0.005) based on publically available databases (1000 Genomes Project (<http://www.1000genomes.org/>), NHLBI Grand Opportunity Exome Sequencing Project (<https://esp.gs.washington.edu/drupal/>), and the Exome Aggregation Consortium data (<http://exac.broadinstitute.org/)>) were filtered out. The pathogenic effect of rare variants identified in FH genes was established using *in silico* mutation prediction tools: PolyPhen-2([5](#_ENREF_5)), SIFT([6](#_ENREF_6)) and Mutation Taster([7](#_ENREF_7)), and previously published evidence available in the University College London FH Mutation Database (<http://www.ucl.ac.uk/ldlr/Current/>).

**SUPPLEMENTARY RESULTS**

Rare variants that were identified by the deep targeted sequencing were also present in WGS data, and vice versa. The targeted sequencing identified five mutation carriers, three of them in samples not included in the UK10K cohort. Three of the mutations were detected by the *standard* variant calling methods, whereas ‘relaxing’ the variant quality filtering and applying the *sensitive* variant calling pipeline detected one additional *LDLR* mutation (p.(Phe241Ser)). A further one mutation carrier was found by WGS, among children that were not sequenced by the targeted sequencing method, whereas one *LDLR* mutation (p.(Arg633Cys)) and one *APOB* mutation (p.(Arg3527Gln)) were found by both assays.

**SUPPLEMENTARY LIMITATIONS**

Technical limitations of the study include the insufficient sequencing read-depth coverage generated by the targeted sequencing of five exons in the *LDLRAP1* gene, in which FH mutations cause the recessive form of the disease and are very rare, and in exon 2 of the *APOB* gene, where, so far, no FH-causing mutations have been reported (**Figure S1)**. Regions with read-depth lower than 15x could not be analysed.

We were unable to confirm by Sanger sequencing one mutation (*APOB* (p.(Arg3527Gln))) identified by WGS because of unavailability of DNA.

**Table S1.**

Baseline characteristics of analysed ALSPAC children (n=5083) with or without sequencing data at their nine years clinic visit. Variables were compared using two sample t-test. BMI, waist and hip circumference were not normally distributed and are presented as geometric means with an approximate standard deviation (SD).

| **Cohort** | **Whole (not sequenced) ALSPAC (n=3571)** | **Sequenced ALSPAC (n=1512)** |  |
| --- | --- | --- | --- |
| Male (%) | 1815 (50.8) | 755(50) |  |
| **Variable** | **Mean (SD)** | **Mean (SD)** | ***p* value** |
| LDL (mmol/L) | 2.35 (0.58) | 2.35 (0.66) | 0.95 |
| TC (mmol/L) | 4.27 (0.64) | 4.27 (0.74) | 0.84 |
| TG (mmol/L) | 1.13 (0.57) | 1.15 (0.58) | 0.35 |
| HDL (mmol/L) | 1.4 (0.31) | 1.4 (0.31) | 0.88 |
| Non-HDL (mmol/L) | 2.87 (0.63) | 2.88 (0.73) | 0.75 |
| ApoA1 (mg/dL) | 136.2 (20.0) | 135.9 (20.2) | 0.69 |
| ApoB (mg/dL) | 59.4 (13.1) | 58.7 (13.7) | 0.11 |
| CRP (mg/L) | 0.78 (2.53) | 0.84 (3.1) | 0.51 |
| BMI | 17.5 (2.9) | 17.3 (2.8) | 0.001 |
| Waist circumference (cm) | 62.6 (7.9) | 62.0 (7.4) | 0.001 |
| Hip circumference (cm) | 73.6 (7.1) | 73.0 (6.8) | 0.001 |

LDL= low-density lipoprotein cholesterol; TC= total cholesterol; TG= triglycerides; HDL= high-density lipoprotein cholesterol; non-HDL = (TC - HDL); ApoA1= Apolipoprotein A-I; ApoB= Apolipoprotein B; CRP= C-reactive protein; BMI= body mass index

**Table S2.**

Full characteristics of children identified with an FH mutation or an LDL-modifying variant.

↑ variant associated with increased LDL-C

↓ variant associated with low LDL-C

In addition to clear FH-causing mutations, a rare *PCSK9* variant p.(His553Arg) was found in one of the participants (minor allele frequency (MAF) = 0.004, according to the NHLBI Grand Opportunity Exome Sequencing Project (ESP) (<http://evs.gs.washington.edu/EVS/>)). This variant is predicted to be damaging using conventional prediction programmes, but these cannot distinguish between gain-of-function (FH causing) and loss-of-function (LDL-C lowering) effects on PCSK9 ([^8^](#_ENREF_8)). The p.(His553Arg) variant has been previously associated with higher LDL-C in a small study of Black Americans ([^9^](#_ENREF_9)), however no family co-segregation studies have been performed, therefore because of the lack of sufficient evidence we did not include this variant as FH-causing in this study. Two other children with LDL-C in the 22nd and 26th percentiles were found to have a well-known LDL-C-lowering loss-of-function variant in *PCSK9* (p.Arg46Leu)([^10^](#_ENREF_10), [^11^](#_ENREF_11)^) (MAF=0.01)^.

**Table S3.**

Comparison of baseline characteristics (at nine years of age) between FH mutation positive and FH mutation negative individuals (*p* values for either χ² or two sample t-test).

|  | **Mutation Positive (n=6)** | **Mutation Negative (n=1506)** | ***p* value** |
| --- | --- | --- | --- |
| **Male (%)** | 4 (66) | 748 (50) | 0.8 |
| **LDL (mmol/L)** | 4.72 (1.35) | 2.34 (3.14) | 0.02 |
| **TC (mmol/L)** | 6.34 (1.32) | 4.27 (0.72) | 0.03 |
| **TG (mmol/L)** | 0.91 (0.3) | 1.15 (0.58) | 0.1 |
| **HDL (mmol/L)** | 1.2 (0.42) | 1.4 (0.31) | 0.3 |
| **Non-HDL-C (mmol/L)** | 5.05 (1.25) | 2.87 (0.71) | 0.01 |
| **ApoA1 (mg/dL)** | 124.4 (25) | 135.9 (20) | 0.4 |
| **ApoB (mg/dL)** | 104.3 (30) | 58.5 (13) | 0.025 |
| **CRP (mg/L)** | 1.3 (1.78) | 0.83 (3.14) | 0.6 |
| **BMI** | 20 (3.6) | 17.5 (2.7) | 0.2 |
| **Waist circumference (cm)** | 69.4 (12.3) | 62.4 (7.3) | 0.3 |
| **Hip circumference (cm)** | 77.6 (9.4) | 73.3 (6.8) | 0.4 |

**Table S4.**

The biochemical screening for FH based on A. LDL-C, B. TC and C. non-HDL-C levels at nine years of age in ALSPAC using lower cut-offs, previously estimated to give 0.5% and 1% FPR([12](#_ENREF_12)). To examine the performance of measuring non-HDL-C (=TC *minus* HDL-C) as a screening tool for FH we assumed that non-HDL-C is approximately 1.24xLDL-C, which was supported by previous studies([13](#_ENREF_13), [14](#_ENREF_14)). We therefore multiplied the previously proposed LDL-C cut-offs by 1.24, which produced non-HDL-C cut-offs of: 5.27mmol/L, 4.76mmol/L and 4.53mmol/L.

Column I contains screening evaluation using data from sequenced ALSPAC participants only. Column II contains results based on the data being extrapolated to the whole cohort of 5083 ALSAPC participants, assuming that there were no more FH mutations present in the not-sequenced participants. In column III are results after correcting for verification bias, and in column IV are results after applying correction for verification bias and for misclassification based on a reduced sensitivity of NGS methods (90%).

A.

| **LDL-C** | **I** | **II** | **III** | **IV** | **As in Wald et al.** |
| --- | --- | --- | --- | --- | --- |
| **1·66 MoM (3.84 mmol/L)** | |  |  |  |  |
| **DR (95%CI)** | 83% (35.9 to 99.6) | 83% (35.9 to 99.6) | 62.5% (24.5 to 91.5) | 66.7% (29.9 to 92.5) | 93% (89 to 96) |
| **FPR (95%CI)** | 1.53% (1.08 to 2.44) | 1.12% (0.85 to 1.45) | 1.1% (0.8 to1.4) | 1.1% (0.8 to 1.4) | 0.5% |
| **PPV (95%CI)** | 17.8% (5.6 to 34.7) | 8.06% (2.7 to 17.8) | 8.2% (2.7 to 18.1) | 9.8% (3.7 to 20.2) | NA |
| **NPV (95%CI)** | 99.9% (99.6 to 100) | 99.9% (99.9 to 100) | 99.9% (99.8 to 100) | 99.9% (99.8 to 100) | NA |
| **OAPR (95%CI)** | 1:5 (0.07 to 0.41) | 1:11 | 5:56 (0.03 to 0.20) | 6:55 (0.04 to 0.22) | NA |
| **1·58 MoM (3.65 mmol/L)** | |  |  |  |  |
| **DR (95%CI)** | 83% (35.9 to 99.6) | 83% (35.9 to 99.6) | 62.5% (24.5 to 91.5) | 62.5% (24.5 to 91.5) | 96% (92 to 98) |
| **FPR (95%CI)** | 2.25% (1.62 to 3.22) | 1.95% (1.59 to 2.37) | 1.8% (1.4 to 2.2) | 1.8% (1.4 to 2.2) | 1% |
| **PPV (95%CI)** | 12.8% (4.2 to 26.8) | 4.8% (1.6 to 10.9) | 5.3% (1.7 to 1.19) | 6.3% (2.4 to 13.2) | NA |
| **NPV (95%CI)** | 99.9% (99.6 to 100) | 99.9% (99.9 to 100) | 99.9% (99.8 to 100) | 99.9% (99.8 to 100) | NA |
| **OAPR (95%CI)** | 1:7 (0.05 to 0.3) | 1:20 | 5:90 (0.18 to 0.13) | 5:89 (0.18 to 0.13) | NA |

B.

| **TC** | **I** | **II** | **III** | **IV** | **As in Wald et al.** |
| --- | --- | --- | --- | --- | --- |
| **1·42 MoM (6.01 mmol/L)** | |  |  |  |  |
| **DR (95%CI)** | 66.6% (22.3 to 95.7) | 66.7% (22.3 to 95.7) | 50% (15.7 to 84.3) | 50% (15.7 to 84.3) | 94% (91 to 97) |
| **FPR (95%CI)** | 1.2% (0.7 to 1.9) | 0.71% (0.5 to 0.98) | 0.7% (0.5 to 1.0) | 0.7% (0.5 to 1.0) | 0.5% |
| **PPV (95%CI)** | 18.2% (5.2 to 40.3) | 10% (2.8 to 23.7) | 10.3% (2.9 to 24.2) | 10.3% (2.9 to 24.2) | NA |
| **NPV (95%CI)** | 99.9% (99.5 to 100) | 99.9% (99.9 to 100) | 99.9% (99.8 to 100) | 99.9% (99.8 to 100) | NA |
| **OAPR (95%CI)** | 1:5 | 1:9 | 4:35 (0.03 to 0.27) | 4:35 (0.03 to 0.27) | NA |
| **1·37 MoM (5.80 mmol/L)** | |  |  |  |  |
| **DR (95%CI)** | 67% (22.3 to 95.7) | 66.7% (22.3 to 95.7) | 50% (15.7 to 84.3) | 50% (15.7 to 84.3) | 96% (93 to 98) |
| **FPR (95%CI)** | 1.86% (1.2 to 2.7) | 1.36% (1.06 to 1.36) | 1.4% (1.1 to 1.7) | 1.4% (1.1 to 1.7) | 1% |
| **PPV (95%CI)** | 12.5% (3.5 to 29.0) | 5.5% (1.5 to 13.4) | 5.5% (1.5 to 13.4) | 5.5% (1.5 to 13.4) | NA |
| **NPV (95%CI)** | 99.9% (99.5 to 100) | 99.9% (99.9 to 100) | 99.9% (99.8 to 100) | 99.9% (99.8 to 100) | NA |
| **OAPR (95%CI)** | 1:7 | 1:17 | 4:69 (0.02 to 0.14) | 4:69 (0.02 to 0.14) | NA |

C.

| **Non-HDL-C** | **I** | | **II** | | **III** | | **IV** | | **As in Wald et al.** |  |
| --- | --- | --- | --- | --- | --- | --- | --- | --- | --- | --- |
| **5.21 mmol/L (= 4.25mmol/L of LDL-C x 1.24)** | | | |  | |  | |  |  |
| **DR (95%CI)** | | 33.3% (4.3 to 77.7) | | 33.3% (4.3 to 77.7) | | 25% (3.2 to 65.1) | | 22.2% (2.8 to 60.0) | NA |
| **FPR (95%CI)** | | 0.6% (0.3 to 1.1) | | 0.2% (0.08 to 0.34) | | 0.2% (0.1 to 0.4) | | 0.2% (0.1 to 0.4) | NA |
| **PPV (95%CI)** | | 18.2% (2.3 to 51.8) | | 18.2% (2.3 to 51.8) | | 15.4% (1.9 to 45.4) | | 15.4% (1.9 to 45.4) | NA |
| **NPV (95%CI)** | | 99.7% (99.3 to 99.9) | | 99.9% (99.8 to 100) | | 99.8% (99.6 to 99.9) | | 99.9% (99.7 to 99.9) | NA |
| **OAPR (95%CI)** | | 02:09 | | 02:09 | | 2:11 (2.3 to 51.8) | | 2:11 (2.3 to 51.8) | NA |
| **4.76 mmol/L (=3.84mmol/L of LDL-Cx1.24)** | | |  | |  | |  | |  |
| **DR (95%CI)** | 66.7% (22.3 to 95.7) | | 66.7% (22.3 to 95.7) | | 50% (15.7 to 84.3) | | 50% (15.7 to 84.3) | | NA |
| **FPR (95%CI)** | 1.13% (0.66 to 1.8) | | 0.3% (0.2 to 0.5) | | 0.6% (0.4 to 0.9) | | 0.6% (0.4 to 0.9) | | NA |
| **PPV (95%CI)** | 19.1% (5.5 to 41.9) | | 19.1% (5.5 to 41.9) | | 11.4% (3.2 to 26.7) | | 11.4% (3.2 to 26.7) | | NA |
| **NPV (95%CI)** | 99.9% (99.5 to 100) | | 100% (99.9 to 100) | | 99.9% (99.8 to 100) | | 99.9% (99.8 to 100) | | NA |
| **OAPR (95%CI)** | 04:17 | | 04:17 | | 4:31 (3.6 to 29.8) | | 4:31 (3.6 to 29.8) | | NA |
| **4.53 mmol/L (=3.65mmol/L of LDL-C x 1.24)** | | | | |  | |  | |  |
| **DR (95%CI)** | 83.3% (35.9 to 99.6) | | 83.3% (35.9 to 99.6) | | 62.5% (25.4 to 91.5) | | 66.7% (29.9 to 92.5) | | NA |
| **FPR (95%CI)** | 1.46% (0.92 to 2.2) | | 0.4% (0.3 to 0.7) | | 1.0% (0.7 to 1.3) | | 0.9% (0.7 to 1.3) | | NA |
| **PPV (95%CI)** | 18.5% (6.3 to 38.1) | | 18.5% (6.3 to 38.1) | | 9.3% (3.1 to 20.3) | | 11.1% (4.2 to 22.6) | | NA |
| **NPV (95%CI)** | 99.9% (99.6 to 100) | | 100% (99.9 to 100) | | 99.9% (99.8 to 100) | | 99.9% (99.8 to 100) | | NA |
| **OAPR (95%CI)** | 05:22 | | 05:22 | | 5:49 (3.3 to 22.2) | | 6:48 (12.5 to 25.2) | | NA |

**Table S5.**

Mutation positive and mutation negative individual counts used for estimating the screening performance of lipid measurements to identify FH mutation carriers.

|  | **LDL-C<4.25** | | **LDL-C>=4.25** | |
| --- | --- | --- | --- | --- |
| **TC (mmol/L)** | **Mutation Positive (n=1)** | **Mutation Negative (n=1494)** | **Mutation Positive (n=5)** | **Mutation Negative (n=12)** |
| **1.53 MoM** | | | **1.53 MoM** | |
| **<6.47** | 1 | 1491 | 3 | 2 |
| **>=6.47** | 0 | 3 | 2 | 10 |
| **1.42 MoM** | | | **1.42 MoM** | |
| **<6.01** | 1 | 1487 | 1 | 1 |
| **>=6.01** | 0 | 7 | 4 | 11 |
| **1.37 MoM** | | | **1.37 MoM** | |
| **<5.80** | 1 | 1477 | 1 | 1 |
| **>=5.80** | 0 | 17 | 4 | 11 |
| **non-HDL-C (mmol/L)** | **Mutation Positive (n=1)** | **Mutation Negative (n=1494)** | **Mutation Positive (n=5)** | **Mutation Negative (n=12)** |
|  | | |  | |
| **<5.27** | 1 | 1493 | 3 | 4 |
| **>=5.27** | 0 | 1 | 2 | 8 |
|  | | |  | |
| **<4.76** | 1 | 1488 | 1 | 1 |
| **>=4.76** | 0 | 6 | 4 | 11 |
|  | | |  | |
| **<4.53** | 1 | 1483 | 0 | 0 |
| **>=4.53** | 0 | 11 | 5 | 12 |

|  | **LDL-C<4.25** | | **LDL-C>=4.25** | |
| --- | --- | --- | --- | --- |
| **TC (mmol/L)** | **Mutation Positive (n=1)** | **Mutation Negative (n=1494)** | **Mutation Positive (n=5)** | **Mutation Negative (n=12)** |
| **1.53 MoM** | | | **1.53 MoM** | |
| **<6.47** | 1 | 1491 | 3 | 2 |
| **>=6.47** | 0 | 3 | 2 | 10 |
| **1.42 MoM** | | | **1.42 MoM** | |
| **<6.01** | 1 | 1487 | 1 | 1 |
| **>=6.01** | 0 | 7 | 4 | 11 |
| **1.37 MoM** | | | **1.37 MoM** | |
| **<5.80** | 1 | 1477 | 1 | 1 |
| **>=5.80** | 0 | 17 | 4 | 11 |
| **non-HDL-C (mmol/L)** | **Mutation Positive (n=1)** | **Mutation Negative (n=1494)** | **Mutation Positive (n=5)** | **Mutation Negative (n=12)** |
|  | | |  | |
| **<5.27** | 1 | 1493 | 3 | 4 |
| **>=5.27** | 0 | 1 | 2 | 8 |
|  | | |  | |
| **<4.76** | 1 | 1488 | 1 | 1 |
| **>=4.76** | 0 | 6 | 4 | 11 |
|  | | |  | |
| **<4.53** | 1 | 1483 | 0 | 0 |
| **>=4.53** | 0 | 11 | 5 | 12 |

**Table S6.**

True positives, false positives, false negatives and true negatives values used for the estimation of the FH screening performance using the top LDL-C cut-point of 1.84MoM based on: A. sequenced sub-sample (n=1512), B. extrapolated to the whole cohort (n=5083), C. extrapolated to the whole cohort and adjusted for verification bias, D. extrapolated to the whole cohort and adjusted for verification and misclassification biases.

| **A.** |  |  |  |
| --- | --- | --- | --- |
| **LDL-C (1.84MoM)** | **Mut +ve** | **Mut -ve** | **Total** |
| **Test +ve** | 5 | 12 | 17 |
| **Test -ve** | 1 | 1494 | 1495 |
| **Total** | 6 | 1506 | 1512 |
| **B.** |  |  |  |
| **LDL-C (1.84MoM)** | **Mut +ve** | **Mut -ve** | **Total** |
| **Test +ve** | 5 | 12 | 17 |
| **Test -ve** | 1 | 5065 | 5066 |
| **Total** | 6 | 5077 | 5083 |
| **C.** |  |  |  |
| **LDL-C (1.84MoM)** | **Mut +ve** | **Mut -ve** | **Total** |
| **Test +ve** | 5 | 12 | 17 |
| **Test -ve** | 3 | 5063 | 5066 |
| **Total** | 8 | 5075 | 5083 |
| **D.** |  |  |  |
| **LDL-C (1.84MoM)** | **Mut +ve** | **Mut -ve** | **Total** |
| **Test +ve** | 6 | 11 | 17 |
| **Test -ve** | 3 | 5063 | 5066 |
| **Total** | 9 | 5074 | 5083 |

**Figure S1.**

Targeted sequencing gene coverage analysis. On the x axis are exons for each of the sequenced genes: A. *LDLR*, B. *PCSK9*, C. *APOB*, D. *LDLRAP1*. Y axis shows the average read depth on a log scale. Dashed red line indicates the depth of 30x that was used in the *standard* variant calling pipeline, whereas the green dashed line marks the 15x read depth used in the *sensitive* variant calling pipeline.


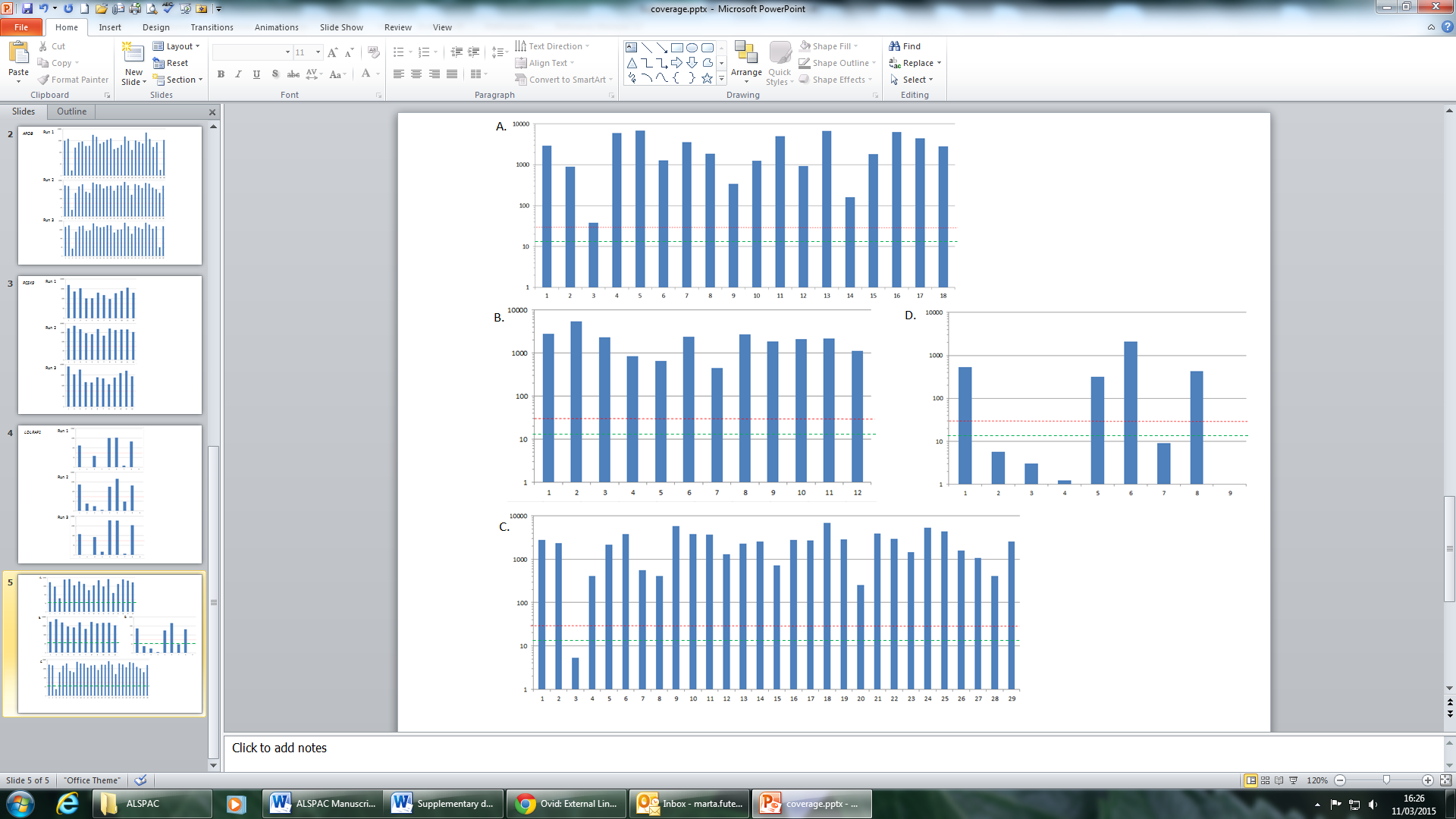


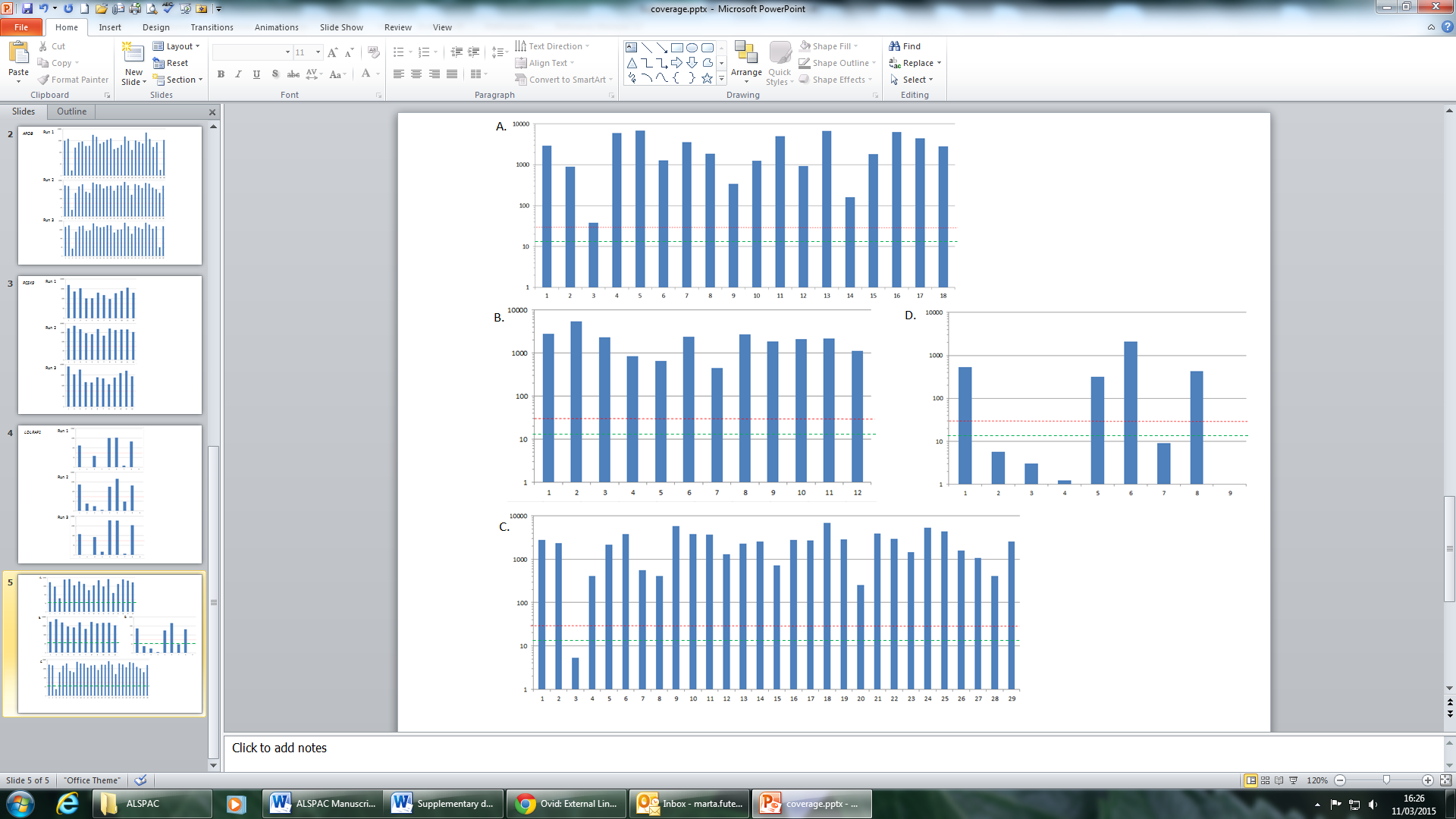


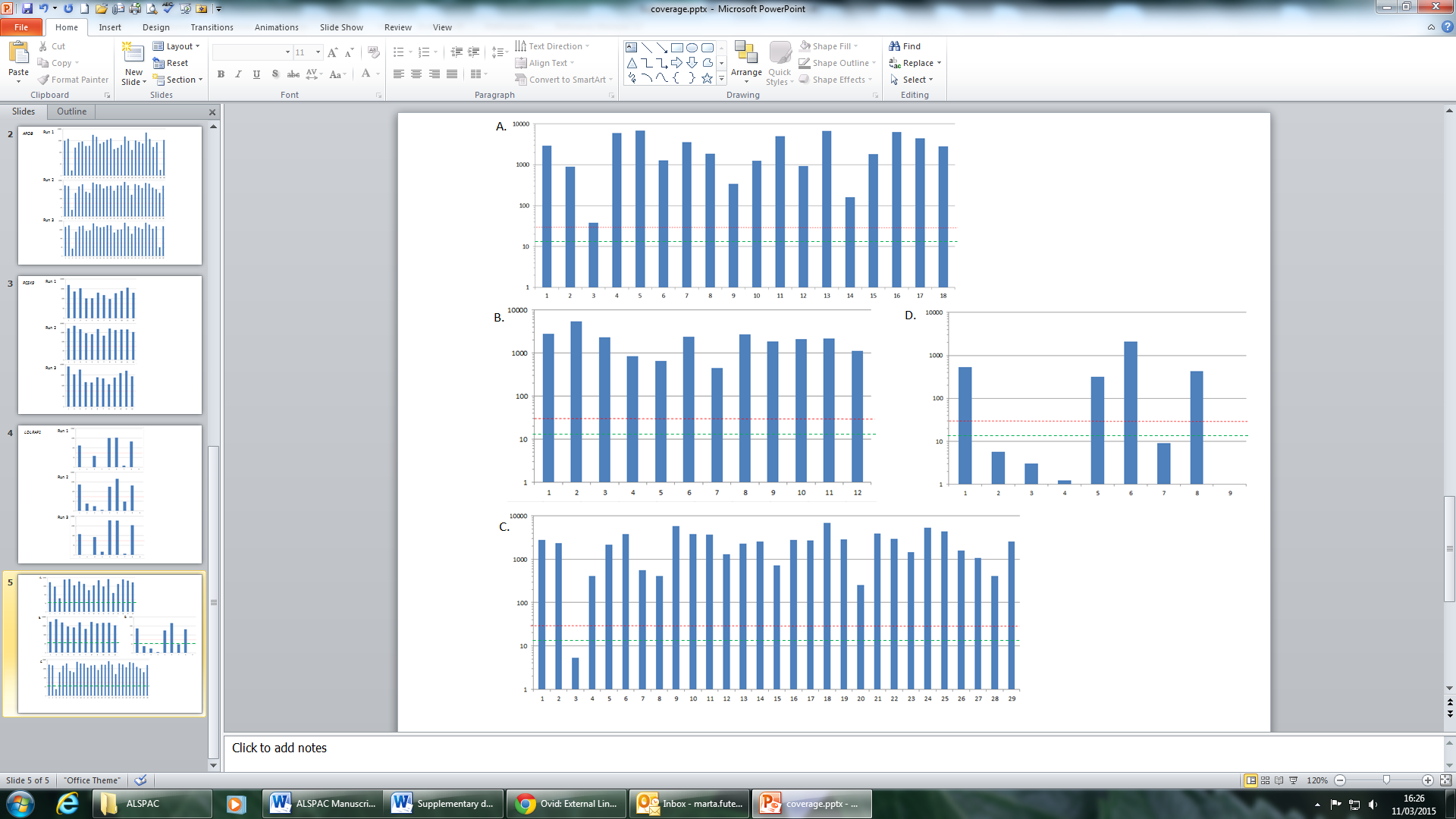


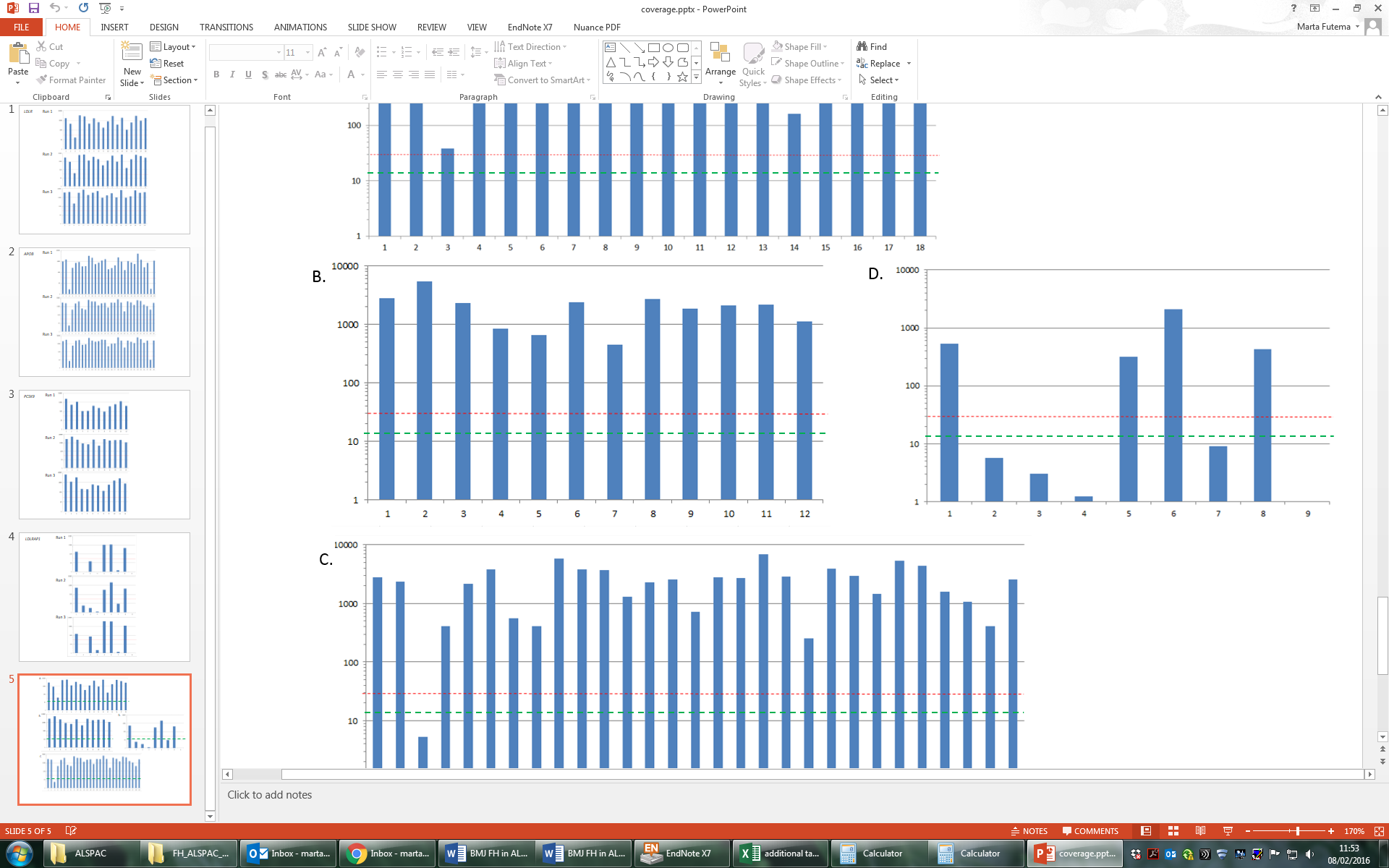


**Figure S2.**

Distribution of six SNP LDL-C genetic risk score in ALSPAC children. The mean score (SD) was 0.62 (0.23), which did not differ from previously studied Whitehall II population 0.63 (0.22)) ([15](#_ENREF_15)).

Six SNP score

Percent

**Figure S3.**

The interpolation of a NGS for samples from all children who screen positive on the basis of an LDL-C above the diagnostic threshold, would eliminate the false positives on the assumption that the DR and FPR of NGS that are close to 100% and 0% respectively and reduce the rate of misclassification among the parental generation, because parents of children with false positive biochemistry screening would be eliminated from the screening. Illustration based on a hypothetical population of 10,000 children.


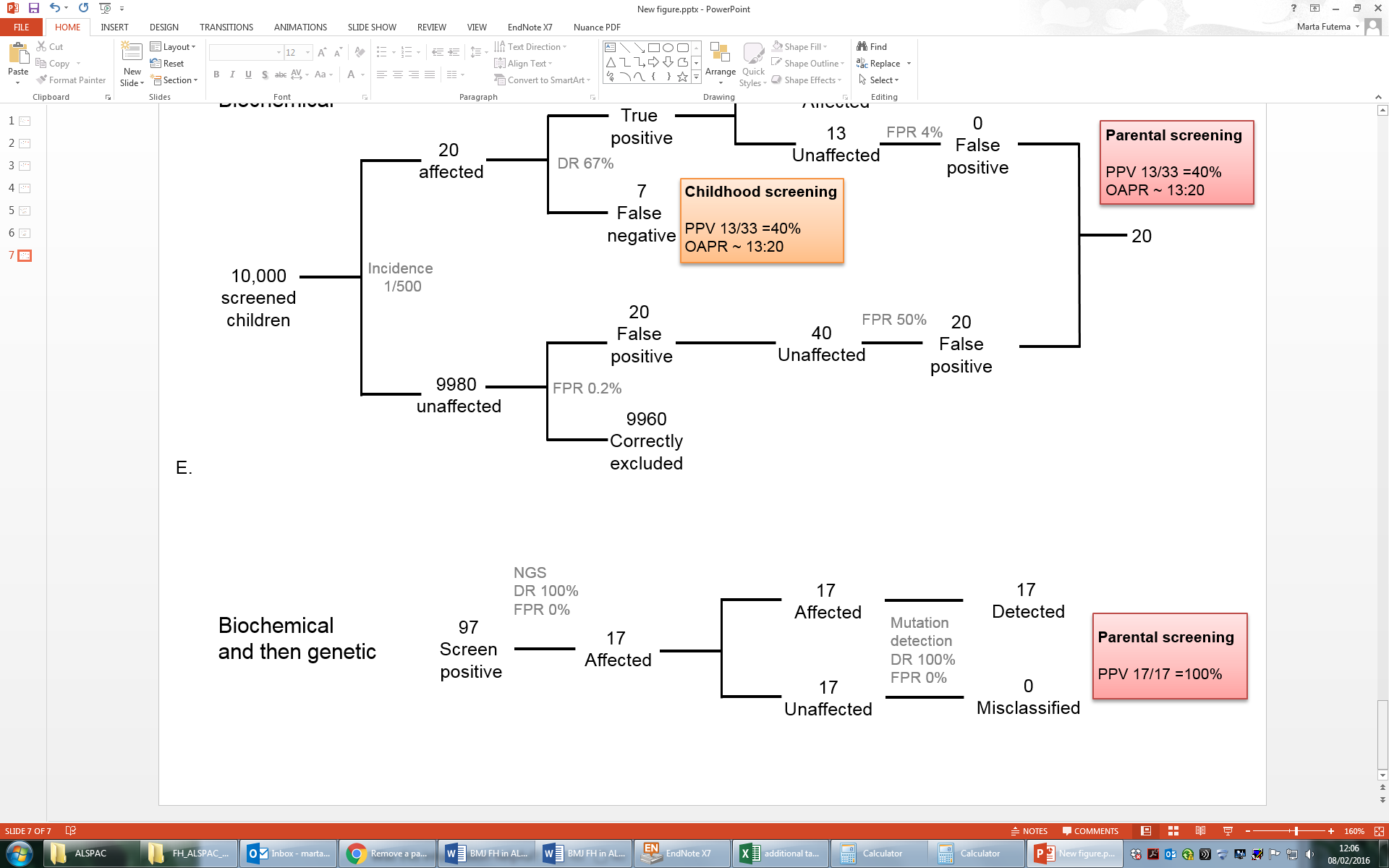


**SUPPLEMENTARY REFERENCES:**

1. Boyd A, Golding J, Macleod J, Lawlor DA, Fraser A, Henderson J, et al. Cohort Profile: the 'children of the 90s'--the index offspring of the Avon Longitudinal Study of Parents and Children. International journal of epidemiology. 2013;42(1):111-27.

2. Timpson NJ, Walter K, Min JL, Tachmazidou I, Malerba G, Shin SY, et al. A rare variant in APOC3 is associated with plasma triglyceride and VLDL levels in Europeans. Nature communications. 2014;5:4871.

3. Consortium UK, Walter K, Min JL, Huang J, Crooks L, Memari Y, et al. The UK10K project identifies rare variants in health and disease. Nature. 2015;526(7571):82-90.

4. Plagnol V, Curtis J, Epstein M, Mok K, Stebbings E, Grigoriadou S, et al. A robust model for read count data in exome sequencing experiments and implications for copy number variant calling. Bioinformatics. 2012.

5. Adzhubei IA, Schmidt S, Peshkin L, Ramensky VE, Gerasimova A, Bork P, et al. A method and server for predicting damaging missense mutations. Nat Methods. 2010;7(4):248-9.

6. Ng PC, Henikoff S. SIFT: Predicting amino acid changes that affect protein function. Nucleic Acids Res. 2003;31(13):3812-4.

7. Schwarz JM, Rodelsperger C, Schuelke M, Seelow D. MutationTaster evaluates disease-causing potential of sequence alterations. Nat Methods. 2010;7(8):575-6.

8. Leigh SE, Leren TP, Humphries SE. Commentary PCSK9 variants: A new database. Atherosclerosis. 2009;203(1):32-3.

9. Kotowski IK, Pertsemlidis A, Luke A, Cooper RS, Vega GL, Cohen JC, et al. A spectrum of PCSK9 alleles contributes to plasma levels of low-density lipoprotein cholesterol. Am J Hum Genet. 2006;78(3):410-22.

10. Abifadel M, Varret M, Rabes JP, Allard D, Ouguerram K, Devillers M, et al. Mutations in PCSK9 cause autosomal dominant hypercholesterolemia. Nat Genet. 2003;34(2):154-6.

11. Scartezini M, Hubbart C, Whittall RA, Cooper JA, Neil AH, Humphries SE. The PCSK9 gene R46L variant is associated with lower plasma lipid levels and cardiovascular risk in healthy U.K. men. Clin Sci (Lond). 2007;113(11):435-41.

12. Wald DS, Bestwick JP, Wald NJ. Child-parent screening for familial hypercholesterolaemia: screening strategy based on a meta-analysis. Bmj. 2007;335(7620):599.

13. Robinson JG, Wang S, Smith BJ, Jacobson TA. Meta-analysis of the relationship between non-high-density lipoprotein cholesterol reduction and coronary heart disease risk. Journal of the American College of Cardiology. 2009;53(4):316-22.

14. Board JBS. Joint British Societies' consensus recommendations for the prevention of cardiovascular disease (JBS3). Heart. 2014;100 Suppl 2:ii1-ii67.

15. Talmud PJ, Shah S, Whittall R, Futema M, Howard P, Cooper JA, et al. Use of low-density lipoprotein cholesterol gene score to distinguish patients with polygenic and monogenic familial hypercholesterolaemia: a case-control study. Lancet. 2013;381(9874):1293-301.
